# Effect of a Targeted Women's Health Intervention in an Inner-City Emergency Department

**DOI:** 10.1155/2011/543493

**Published:** 2011-12-10

**Authors:** Debra Houry, Abigail Hankin, Jill Daugherty, L. Shakiyla Smith, Nadine Kaslow

**Affiliations:** ^1^Department of Emergency Medicine, Emory University, Atlanta, GA 30322, USA; ^2^Rollins School of Public Health, Emory University, Atlanta, GA 30322, USA; ^3^Department of Psychiatry and Behavioral Sciences, Emory University, Atlanta, GA 30322, USA

## Abstract

*Objective.* To evaluate the effect of an Emergency Department (ED) based, educational intervention for at-risk health behaviors. *Methods.* A randomized trial over a one-year period. African American women, aged 21–55, presenting to the ED waiting room were eligible. Each participant took a computer-based survey on health risk behaviors. Participants who screened positive on any of four validated scales (for IPV, nicotine, alcohol, or drug dependence) were randomized to standard information about community resources (control) or to targeted educational handouts based upon their screening results (intervention). Participants were surveyed at 3 months regarding contacts with community resources and harm-reduction actions. *Results.* 610 women were initially surveyed; 326 screened positive (13.7% for IPV, 40.1% for nicotine addiction, 26.6% for alcohol abuse, and 14.4% for drug abuse). 157 women were randomized to intervention and 169 to control. Among women who completed follow-up (*n* = 71), women in the Intervention Group were significantly more likely to have contacted local resources (37% versus 9%, *P* = 0.04) and were more likely to have taken risk-reducing action (97% versus 79%, *P* = 0.04). *Conclusion.* Targeted, brief educational interventions may be an effective method for targeting risk behaviors among vulnerable ED populations.

## 1. Introduction

Although African American women represent only 12.7% of women in the United States, they are disproportionately medically underserved [1]. For example, African American women are over 30% more likely than white women to die of cancer [2], and low-income African American women are more likely than higher income or nonminority women to report that they lack health insurance or a regular provider and were less likely to report that they had been given appropriate health risk counseling when seeing health care providers [3]. Over the past two decades the African American-Caucasian gap has not narrowed with respect to access to and use of health services [4]. Emergency departments (EDs) are used for medical treatment by those with limited access to care, including low-income people of color, and thus are an opportune place to focus on interventions and referrals for public health issues such as violence and substance use.

African American women are at higher risk than women from other demographic groups for many health-related risk factors, including intimate partner violence (IPV) victimization [5], as well as alcohol, drug, and nicotine dependence. While IPV is of concern among all ethnic groups, research suggests that African American couples are at higher risk of IPV, even after controlling for socioeconomic status and alcohol use [6]. Some studies also suggest that African American women are more likely than people from other ethnic groups to experience severe injuries as a result of IPV [7], and murder by an intimate partner is the leading cause of death among African American women between the ages of 15 and 45 [8]. Furthermore, African American women may experience more severe mental health consequences due to IPV victimization [9].

In terms of substance use, African American women are almost three times more likely to be heavy drinkers than their Caucasian counterparts [10]. Additionally, compared to other ethnic groups, African Americans endorse the highest incidence of illicit drug use. Results from SAMHSA's 2007 National Survey on Drug Use and Health found that 9.5% of African Americans aged 12 and older reported current illicit drug use, as compared with 8.2% of Whites and 6.6% of Latinos [11]. Urban African American women have high levels of crack use compared to women from other ethnicities [12]. Although smoking rates in African American women are on the decline, 22% continue to smoke, comparable with rates in Caucasian women (26%) [13]. Furthermore, minority status appears to increase risk of cardiac and cardiovascular consequences of smoking and is also associated with lower rates of access to and use of smoking cessation programs [3]. Low-income African American women were more likely to smoke than those with moderate- to high-income levels, 22.9% versus 15.7% [14].

Furthermore, these individual risk factors are believed to have a cumulative effect [15], each increasing women's vulnerability to other mental and physical health risks and together have a multiplicative effect on health [16]. For example, recent studies have shown that women who are victims of IPV are at increased risk for a wide range of chronic health problems, such as stomach ulcers, migraines, and chronic pelvic pain, as well as more likely to report generally poor mental and physical health [17]. Additionally, the adverse consequences of IPV extend beyond the direct victim and are correlated with developmental delays; increased rates of problems at school and behavioral problems; low self-esteem in children exposed to IPV [18].

The higher rates of IPV and substance misuse among African American women and the access of EDs among minority populations [19, 20] create an opportunity to utilize the ED as a venue for education around preventive care and thus as a mechanism for improving the health of the communities served [21]. In response to the need and opportunity for targeted screening and intervention in this population, we developed a program of computer-based screening linked with tailored resources and brief, culturally-competent educational materials. Prior ED-based studies involving this population have shown that computer-based screening for IPV and mental health indices is safe, effective, and accurate [22]. We hypothesized that women randomized to the computer-based educational intervention would be more likely to contact resources and decrease their health risk behaviors than women in the control group.

## 2. Methods

### 2.1. Study Design

We conducted a prospective randomized trial of African American women presenting to an academic, urban ED. All African American women, aged 21–55, who presented to the emergency department waiting room during study hours, were eligible for participation, regardless of chief complaint. Patients were excluded if they were non-English speakers, acutely intoxicated, actively psychotic, critically ill/required immediate medical attention, or unable to complete a 15-minute questionnaire. The study was approved by our university's Institutional Review Board as well as the hospital research oversight committee.

### 2.2. Setting

The study was conducted in the waiting room of a public hospital in a large southern city. The hospital serves a largely inner-city and medically indigent population, with a predominantly African American population (92%). The ED has 57 emergency medicine residents supervised by 70 emergency medicine faculty physicians. Total patient volume is approximately 105,000 patient visits per year.

### 2.3. Procedure

A research assistant (RA) determined eligibility at the time of patient registration by reviewing the triage note, then approached patients to participate in a general health survey, and provided instructions for using the computer after receiving informed consent. The computer-based survey included questions about demographics, general health, and health behaviors and included previously validated screening tools for IPV and nicotine, drug, and alcohol dependence as well as the Beck Depression Inventory; the survey required no more than fifteen minutes of participant time (and employed a skip design, thus often requiring far less time for women who screened negative on most scales). Upon completion of the computerized survey, the computer randomly assigned each participant who screened positive for IPV, alcohol abuse, drug abuse, or cigarette smoking to either the educational intervention Group or Enhanced Usual Care (described below). Randomization was performed via random number generator; the research assistant was alerted regarding survey completion and study group assignment but did not have access to individual patient survey responses.

At three months following the initial screening, participants were asked to return for a followup assessment, which included a computer-based followup screening and a structured, face-to-face assessment. The followup interview contained all of the questions asked in the initial survey, as well as questions specifically inquiring about contact and use of the recommended community resources, as well as whether participants had taken any of the harm-reduction steps recommended in the educational brochures.

### 2.4. Intervention

In preparation for this study, we developed and pilot-tested educational materials on IPV, alcohol and drug abuse, and smoking using focus groups of African American female ED patients. A two-sided handout written for a 5th-grade reading level was given to focus group participants. Their input on readability, cultural issues, and impact was used to update the materials accordingly.

These revised education materials were given to our study participants in the Intervention Group for each health risk behavior for which they screened positive, with each brochure providing facts and resource referrals related to each specific health risk behavior. Additionally, a research assistant would carefully go over the information in the brochures with participants who were assigned to receive one or more brochures. There were four different brochures related to IPV, and nicotine, alcohol, or drug dependence, each with tailored information. For example, the brochure on IPV offered information about unhealthy relationships and referrals for shelters, legal services, and support groups. Participants in the Intervention Group received between one and four brochures based on their responses. Participants in the control group received a brochure on neighborhood clinics in the area.

## 3. Measures

### 3.1. IPV Instrument

The Index of Spouse Abuse (ISA) is a 30-item scale designed to detect IPV in women [23]. There are 2 subscales, the ISA-P, which measures the severity of physical abuse and the ISA-NP, which measures the severity of nonphysical abuse. Questions ask women about their experience of specific physically and emotionally abusive acts by their partner. Each question is answered using a 5-point Likert scale with answers ranging from never to very frequently, with each answer being scored 1 to 5, respectively. A participant's score is based on scoring their answers according to a preset formula. Using reported cut points for the ISA-P of 10 and the cut point for the ISA-NP of 25, the sensitivity is 92.9% and specificity is 90.7%. Both the ISA-P and the ISA-NP are highly reliable,  .94 and  .97, respectively [24]. Both subscales were also found to have high validity, ISA-P  =  .73 and ISA-NP  =  .80 [22]. An ISA-P score of ≥10 or an ISA-NP score of ≥25 was considered a positive screen for IPV [24]. 

### 3.2. Alcohol Abuse Instrument

The TWEAK (Tolerance, Worried, Eye openers, Amnesia, K(C)utdown) scale was used for detecting alcohol abuse. The TWEAK consists of 5 questions, including four yes/no questions and one numerical question (“How many drinks can you hold.”) scored on a 7-point scale [25]. There are two versions of the tolerance question and for this study, “How many drinks can you hold” was used. This version has been found to increase sensitivity scores to 91% and specificity scores to 77% for the overall scale [26].

 A score of ≥2 was used to identify alcohol abusers in our study. This cut point has been suggested by several studies as being the most appropriate cut point to use for females [27] and for patients in the ED [28].

### 3.3. Drug Abuse Instrument

The Drug Abuse Screening Test (DAST20) [22] was used to assess whether participants are abusing drugs. The DAST20 was developed from a longer 28-item scale and was found to correlate almost perfectly with the 28-item scale (*r* = .99) [29]. Patients respond either “yes” or “no” to a series of 20 questions about drug use in the past 12 months. The DAST has been shown to have high internal consistency (.94) and good item-total scale correlations with 89% accuracy in classifying patients according to DSM-III substance abuse diagnosis. The DAST also has high sensitivity of 96% and a specificity of 81% [29]. Scores ≥6 are coded as positive for drug abuse, and a score of ≥16 indicates a severe problem with drug abuse. For the purposes of this study, a score of ≥6 was considered a positive screen for drug abuse [30].

### 3.4. Smoking Instrument

The Hooked on Nicotine Checklist (HONC) was used to assess smoking in our population. The 10-item tool was developed to assess the loss of autonomy over nicotine in adolescents and has been validated for use in adults [31]. The HONC has an internal reliability of 0.83 [32]. The HONC has a sensitivity of 93% and a specificity of 75% [33]. A score of ≥1 is considered a positive screen for smoking [32].

### 3.5. Depression Instrument

The Beck Depression Inventory, II (BDI-II) was used to assess the presence and severity of depressive symptoms [34]. In this study, we used a BDI-II score of 20 or greater, consistent with moderate-to-severe depression, as a positive depression screen. Prior validation studies have established an overall classification rate of 88% using this cut point (sensitivity 71%, specificity 88%) [35]. Results of this screen were not utilized neither for the targeted brief interventions nor for determining study inclusion.

## 4. Data Analysis

We used SAS statistical software (version 9.2, SAS Institute, Cary, North Carolina) for data analyses. We utilized a series of *χ*2 tests to assess bivariate associations between study arm (Intervention Group versus Enhanced Usual Care) and contact with a community resource or harm-reduction action taken. Adjustment for age as a potentially important confounder was achieved through logistic regression where contact with a community resource or harm-reduction action taken was the dependent variable, and study arm and age were independent variables. Age was modeled as a continuous predictor.

## 5. Results

A total of 1250 women were eligible to participate in this project; of these 610 women consented to participate (49%). Of the 610 women who participated, 18 women (3%) chose not to complete the entire survey, and 270 (44.3%) screened negative on all instruments surveyed and thus were not included in the intervention stage of the project.

Of the 610 participants, 322 women (52.8%) screened positive for at least one survey instrument and were randomized to Enhanced Usual Care or the educational intervention “Intervention Group.” There were no between-group differences with respect to marital status, education level, or results of IPV or substance abuse screening, but there was a difference between the groups with respect to age, with women in the Intervention Group being slightly older (*P* < 0.01) (Table 1). 

At three months, of the 322 women who were randomized, 72 (22.3%) participants completed followup screening. Compared with women who completed the followup survey at three months, women were more likely to be lost to followup if they screened negative for alcohol dependence (RR = 0.88) or screened positive for physical IPV (RR = 1.16). An association was not present for women who screened positive for nonphysical IPV, nicotine dependence, drug dependence, or depression. There was no association between randomization to Enhanced Usual Care versus Intervention and patient loss to followup (Table 2). 

Among women who completed the followup survey at three months, women who were in the Intervention Group, in aggregate, were more likely to have “contacted anyone” of the local support agencies than were women in the Enhanced Usual Care Group (OR = 3.55). When broken down by social support service categories (IPV, or nicotine, alcohol, or drug dependence), women in the Intervention Group showed a strong trend towards increased resource contact, although not statistically significant.

Furthermore, when asked about whether they had taken any harm-reduction actions, such as “made a quitting plan” or “cut down on the amount of drugs [they] used”, women in the Intervention Group were more likely to have taken harm-reduction actions than were those in the Enhanced Usual Care group (OR of “taking any harm-reduction action”: 1.22). By intervention category (i.e., IPV or nicotine, alcohol, or drug dependence), women in the Intervention Group were significantly more likely to have taken steps to reduce their alcohol use (OR = 1.78), and women in the Intervention Group showed a trend towards increased probability of taking action to reduce tobacco use (*P* = 0.22).

When controlled for age, previously significant results remained significant and to be quite robust. Members of the Intervention Group remained more likely to “Contact any local organization” (OR: 5.05,  *P* = 0.025), “taking any harm-reduction” (OR: 2.11,  *P* = 0.048). An exception to this finding was the finding that, when controlled for age, women in the Intervention Group continued to show a trend (*P* = 0.061) towards taking action based on alcohol dependence, but this trend did not achieve statistical significance.

## 6. Discussion

In this randomized trial, women who were randomized to receive targeted information based upon the results of computerized screening were over three times more likely to contact local resources and more likely to take one of the harm-reduction actions. In terms of specific risk factors, we found that women who were exposed to the brief educational intervention (i.e., the Intervention Group) were more likely to take recommended harm-reduction actions (such as making a quitting plan or moving out of an unsafe housing situation) if they had screened positive for alcohol abuse, and women who had screened positive for nicotine abuse showed a strong trend towards increased likelihood of contacting recommended community resources. Across all health risk factors, women who had been exposed to the targeted educational intervention were more likely to be positive for “contacting any recommended organization” and/or “taking any recommended action”. We did not find significant differences at the level of individual risk factors other than nicotine or alcohol with respect to the probability of contacting a local resource and/or taking harm-reduction actions (e.g., contacting local domestic violence support agencies, or taking recommended harm reduction steps for substance abuse). The lack of significance at the level of individual risk factors and actions is likely related to the small sample sizes at followup and suggest a need for larger studies with steps taken to optimize followup.

Prior research with women in this population has shown that the transtheoretical (“stages of change”) model can be applied to women who are victims of IPV and who are accessing the ED. In a prior study, the vast majority (95%) of women in our population were found to be in the pre contemplation and contemplation stages [36]. The current study suggests that women in this population may also be largely in the precontemplation and early contemplation stages with respect to other health risk factors such as alcohol/drug abuse and smoking; if this is correct, our findings suggest that a brief, targeted intervention is sufficient to encourage participants to the stages of preparation and even action [37].

A relatively large number of women in both the intervention and the control groups were lost to followup at three months. On further analysis, we found that women were more likely to be lost to followup if they had fewer years of formal education. Women were less likely to be lost to followup if they had screened positive for physical IPV, if they were widows (versus single), or if they screened positive for alcohol dependence. Factors including age, marital status (with the exception of women who were widowed), and screening results for nonphysical IPV or other substances, did not correlate with loss to followup.

Prior ED-based studies have shown that computer-based screening for intimate partner violence is a reliable method of prompting women to disclose emotional and physical abuse [28, 38] as well as substance abuse and depression [39]. Additionally, other researchers have evaluated the role of screening and brief intervention (SBIRT) in the ED, primarily as relateing to alcohol abuse and have found that even brief interactions between at-risk ED patients and trained counselors can lead to a decrease in alcohol use and increased readiness to quit [40, 41]. This literature suggests that an ED visit may represent a “teachable moment” for at-risk patients and that an ED visit may provide the opportunity for screening and intervention with respect to a variety of risk factors. The intervention utilized in the current study is especially promising because it requires a minimum amount of staff time and does not inherently require specially trained staff, allowing for the possibility of application of this approach in resource-poor EDs.

## 7. Limitations

Our study has several limitations. First, a relatively large number of women in both the intervention and the control groups were lost to followup at three months. Women were most likely to be lost to followup if they had fewer years of formal education and if they had initially screened positive for nicotine dependence, whereas women who were IPV victims were least likely to be lost to followup. Though reasons for loss to followup were not known in most cases, contributing factors may include high rates of transience, low income (and thus loss/change of phone lines), and housing instability in this urban, inner-city population may be contributing factors. The high rate of loss to followup may limit our ability to generalize these findings to the larger population of interest, as well as limiting our ability to study specific characteristics that may make women more likely to act based on brief, targeted interventions. However, given the low-cost, low-resource nature of this intervention, finding an effect, even in a segment of the study population, is important and can serve to shape ED-based interventions for women at risk for IPV victimization.

An additional limitation is the survey design, relying on patient self-report at followup to determine whether they had contacted local resources or taken harm-reduction action. This may have led patients to overreport the actions due to social desirability bias. However, we used validated scales, and patients were blinded with respect to their randomization status. Finally, this study focused on a specific at-risk population, and thus results may not be generalizable to other patient groups in different clinical settings.

## 8. Conclusion

In summary, computer-based screening for IPV, or tobacco alcohol and drug dependence, combined with targeted distribution of patient-oriented educational literature, is a promising approach to providing education and resource referrals to ED patients. Further understanding of how best to target such interventions, and how to maximize the efficiency and effectiveness of such interventions is a critical area of research in the field of injury/violence prevention and emergency medicine, given resource limitations in ED environments (particularly inner-city EDs).

## Figures and Tables

**Table 1:** Initial characteristics of patients randomized to the Intervention Group versus Enhanced Usual Care. “Negative” indicates patients who screened negative on all instruments utilized and were not randomized, “Quit” indicates patients who did not complete initial survey. (ISA-P: Index of Spousal Abuse, physical ISA-NP: Index of Spousal Abuse, nonphysical HONC: hooked on nicotine checklist TWEAK: Tolerance, Worried, Eye openers, Amnesia, K(C)utdown (alcohol screening) DAST: Drug Abuse Screening Test BDI: Beck's Depression Inventory). *: indicates *P* < 0.05.

	Enhanced Usual Care (*n* = 155)	Intervention (*n* = 156)	Negative (*n* = 270)	Quit (*n* = 16)	*P* value
Mean age (range)*	37.9 (21–55)	41.5 (21–65)	38.0 (21–62)	39.9 (23–57)	0.0011
Marital status					
Single	69.0%	73.0%	68.9%	75.0%	0.6293
Separated	10.3%	12.2%	7.0%	6.3%	
Divorced	12.9%	9.6%	10.0%	6.3%	
Widowed	1.9%	1.3%	3.3%	0%	
Now married	5.8%	9.6%	10.7%	12.5%	
Education:					
<9th grade	5.8%	8.3%	3.7%	12.5%	0.9063
Some HS	23.9%	25.0%	11.5%	6.3%	
High school	36.8%	38.1%	43.3%	62.5%	
Some college	25.8%	25.0%	28.1%	23.1%	
College	7.7%	10.3%	13.0%	0	
Initial screening:					
ISA-P +	18.1%	25.0%	0	0	0.2206
ISA-NP +	20.6%	25.6%	0	6.3%	0.4405
HONC +	74.8%	80.1%	0	6.3%	0.8373
TWEAK +	45.8%	57.1%	0	12.5%	0.1462
DAST +	22.6%	32.7%	0	12.5%	0.093
BDI +	14.2%	28.2%	0	6.3%	0.9326

**Table 2:** Comparison of characteristics of patients lost to followup versus those not lost, and relative risk of loss to followup. Please see Table 1 for key to abbreviations. *: Indicates  *P* < 0.05.

	Lost + (*n* = 249)	Not lost (*n* = 73)	Relative risk	*P* value
Age	mean: 39.33	mean: 41.26		*P* = 0.1466
Marital status:				
Single	70.7%	63.0%	Ref.	
Separated	10.4%	13.7%	0.9	*P* = 0.2957
Divorced	11.2%	9.6%	1.01	*P* = 0.9220
Widowed*	0.4%	5.5%	0.25	*P* = 0.0089
Now married	7.2%	8.2%	0.95	*P* = 0.6257
Education:				
<9th grade	8.4%	1.4%	1.3	*P* = 0.0238
Some HS	24.5%	20.5%	1.1	*P* = 0.2673
High school*	34.1%	42.5%	Ref.	
Some college*	22.5%	31.5%	0.97	*P* = 0.7143
College*	10.0%	4.1%	1.22	*P* = 0.0734
Screening results:				
ISA-P +*	23.4%	12.3%	1.16	*P* = 0.0424
ISA-NP +	24.9%	16.4%	1.1	*P* = 0.1673
HONC +	76.3%	74.0%	1.03	*P* = 0.6825
TWEAK +*	46.6%	60.3%	0.88	*P* = 0.0397
DAST +	26.1%	28.8%	0.97	*P* = 0.6511
BDI +	26.5%	27.4%	0.99	*P* = 0.8797
Intervention	53.0%	46.6%	1.06	*P* = 0.3332
